# Caffeic Acid Phenethyl Ester Induces *N*-myc Downstream Regulated Gene 1 to Inhibit Cell Proliferation and Invasion of Human Nasopharyngeal Cancer Cells

**DOI:** 10.3390/ijms19051397

**Published:** 2018-05-08

**Authors:** Kun-Chun Chiang, Shih-Wei Yang, Kai-Ping Chang, Tsui-Hsia Feng, Kang-Shuo Chang, Ke-Hung Tsui, Yi-Syuan Shin, Chiu-Chun Chen, Mei Chao, Horng-Heng Juang

**Affiliations:** 1 Zebrafish Center, Department of General Surgery, Chang Gung Memorial Hospital, Keelung 204, Taiwan; robertviolet6292@yahoo.com.tw; 2 Department of Otolaryngology Head and Neck Surgery, Chang Gung Memorial Hospital, Keelung 204, Taiwan; sweeyang@gmail.com; 3 Department of Otolaryngology Head and Neck Surgery, Chang Gung Memorial Hospital Lin-Kou, Kwei-Shan, Tao-Yuan 204, Taiwan; dr.kpchang@gmail.com; 4 School of Nursing, College of Medicine, Chang Gung University, Kwei-Shan, Tao-Yuan 244, Taiwan; thf@mail.cgu.edu.tw; 5 Department of Anatomy, College of Medicine, Chang Gung University, Kwei-Shan, Tao-Yuan 244, Taiwan; D0501301@stmail.cgu.edu.tw; 6 Department of Urology, Chang Gung Memorial Hospital-Linkou, Kwei-Shan, Tao-Yuan 244, Taiwan; t2130@cgmh.org.tw; 7 Department of Medicine, College of Medicine, Chang Gung University, Kwei-Shan, Tao-Yuan 244, Taiwan; a0956910758@gmail.com (Y.-S.S.); a5880018@gmail.com (C.-C.C.); 8 Department of Microbiology and Immunology, College of Medicine, Chang Gung University, Kwei-Shan, Tao-Yuan 244, Taiwan; pa0728@mail.cgu.edu.tw; 9 Department of Hepato-Gastroenterology, Liver Research Center, Chang Gung Memorial Hospital Lin-Kou, Kwei-Shan, Tao-Yuan 244, Taiwan

**Keywords:** CAPE, NDRG1, MAPK, nasopharyngeal cancer, STAT3

## Abstract

Caffeic acid phenethyl ester (CAPE), a bioactive component extracted from propolis, is widely studied due to its anti-cancer effect. Nasopharyngeal carcinoma (NPC) is distinct from other head and neck carcinomas and has a high risk of distant metastases. *N*-myc downstream regulated gene 1 (NDRG1) is demonstrated as a tumor suppressor gene in several cancers. Our result showed that CAPE treatment could repress NPC cell growth, through induction of S phase cell cycle arrest, and invasion. CAPE treatment stimulated NDRG1 expression in NPC cells. NDRG1 knockdown increased NPC cell proliferation and invasion and rendered NPC cells less responsive to CAPE growth-inhibiting effect, indicating CAPE repressed NPC cell growth partly through NDRG1indcution. CAPE treatment increased phosphorylation of ERK, JNK, and p38 in a dose- and time-dependent manner. Pre-treatments by inhibitors of ERK (PD0325901), JNK (SP600125), or p38 (SB201290), respectively, all could partly inhibit the CAPE effect on NDRG1 induction in NPC cells. Further, STAT3 activity was also repressed by CAPE in NPC cells. In summary, CAPE attenuates NPC cell proliferation and invasion by upregulating NDRG1 expression via MAPK pathway and by inhibiting phosphorylation of STAT3. Considering the poor prognosis of NPC patients with metastasis, CAPE could be a promising agent against NPC.

## 1. Introduction

Nasopharyngeal carcinoma (NPC) is one kind of cancer stemming from the nasopharynx epithelium. Although NPC is not the only one cancer coming from the epithelium of the head and neck region, NPC has distinct characteristics as compared to other epithelial tumors in the head and neck [1]. The World Health Organization classifies NPC into two separate categories: type I keratinizing squamous cell carcinoma and type II keratinizing carcinoma, which can be further subdivided into differentiated and undifferentiated subtypes [2]. Though the incidence of NPC is not high, NPC is highly endemic in east and southeast parts of Asia with around 86,000 new cases diagnosed and 50,000 deaths annually worldwide [3,4]. NPC is generally sensitive to both chemotherapy and radiotherapy; however, for recurrent or metastatic NPC, the prognosis is still very poor. Thus, there is much room for improvement in NPC treatment. 

Propolis is a natural product derived from honeybee hives. Propolis has been shown to possess a broad spectrum of pro-healthy functions, including anti-microbial, anti-oxidative, anti-ulcer, and anti-tumor activities, and thus is widely used as an additive in foods and beverages [5]. Caffeic acid phenethyl ester (CAPE) is the most bioactive compound of propolis and has been demonstrated to have anti-oxidant, anti-inflammatory, and anti-tumor properties [6,7]. CAPE can further be shown to be able to inhibit angiogenesis and tumor metastasis, and acts as a potent radiosensitizer in various human cancers, including oral cancer [8,9,10]. So far, a myriad of cancers has been shown to be repressed by CAPE, including breast cancer, melanoma, prostate cancer, oral cancer, head and neck squamous carcinoma, lung cancer, etc. [11,12,13,14,15]. Regarding NPC, study is so far lacking. 

*N*-myc downstream regulated genes (NDRGs) belong to a family of cytosolic proteins, consisting of four members, NDRG1, NDRG2, NDRG3, and NDRG4. NDRG1 and NDRG2, which have been well reported as tumor suppressors for oral and esophageal squamous carcinoma [16,17,18,19]. For NPC, NDRG1 has been demonstrated to suppress cell invasion and EMT (epithelial mesenchymal transition) markers [20]. Previously, our group has proven that CAPE could repress oral cancer cell growth through upregulation of NDRG1 [14].

The objective of this study is to evaluate the effect of CAPE on NPC cells and the related mechanisms, especially the NDRGs expressions. 

## 2. Results

### 2.1. CAPE Inhibits Cell Growth and Invasion in NPC Cells

**Figure 1:**
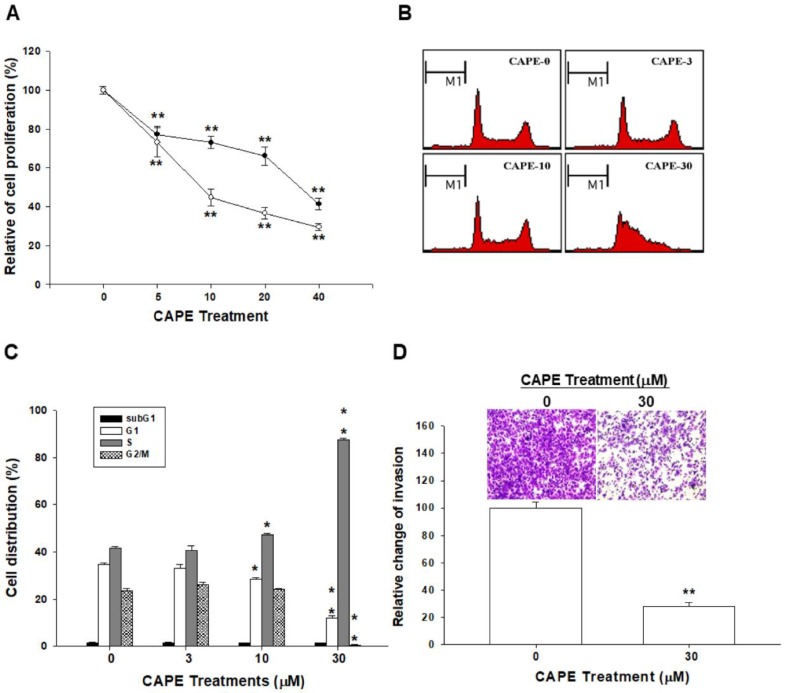
The anti-proliferative and anti-invasive effects of Caffeic acid phenethyl ester (CAPE) in TW04 cells. (**A**) TW04 cells were treated with various concentrations of CAPE for 24 h (●) or 48 h (○) and growth inhibitory effect was determined by the CyQuant cell proliferation assay. The data were presented as the mean percentage (±SE) of cells in each dose of the CAPE-treated group and compared with the control solvent-treated group (0 μM CAPE-treated, *n* = 8). (**B**) The represent DNA histograms of TW04 cells treated by different doses of CAPE for 24 h by standard flow cytometry. (**C**) The data were presented as the mean percentage (±SE) and compared with the control solvent-treated group. (**D**) TW04 cells were treated with CAPE (30 μM) for 24 h and the cell invasive ability was measured by matrigel-invasion assay for another 24 h incubation (top). The quantitative data were expressed as average cell counts/9 fields (bottom). (* *p* < 0.05, ** *p* < 0.01).

To investigate the anti-proliferative effect of CAPE on NPC cells, different doses of CAPE were applied to treat TW04 cells. The CyQuant cell proliferation assay revealed that CAPE repressed TW04 cells growth at the doses from 5 to 40 μM after treatment for 24 or 48 h in the dose- and time-dependent manner (Figure 1A). The flow cytometric analysis revealed that CAPE treatments induced TW04 cell cycle arrest at S phase dose-dependently after 24 h treatment (Figure 1B). CAPE (10–30 μM) treatments induced 6.3–45.9% increases of S phase cells of TW04 cells (Figure 1C). The result of the matrigel invasion assay also showed that CAPE treatment significantly decreased the cell invasion in TW04 cells (Figure 1D, top). Quantitative analysis indicated a significant 71.6% reduction of TW04 cell invasion after 30 μM CAPE treatment for 24 h in comparison with the control cells (Figure 1D, bottom).

### 2.2. CAPE Upregulates NDRG1 Expression in NPC Cells

**Figure 2:**
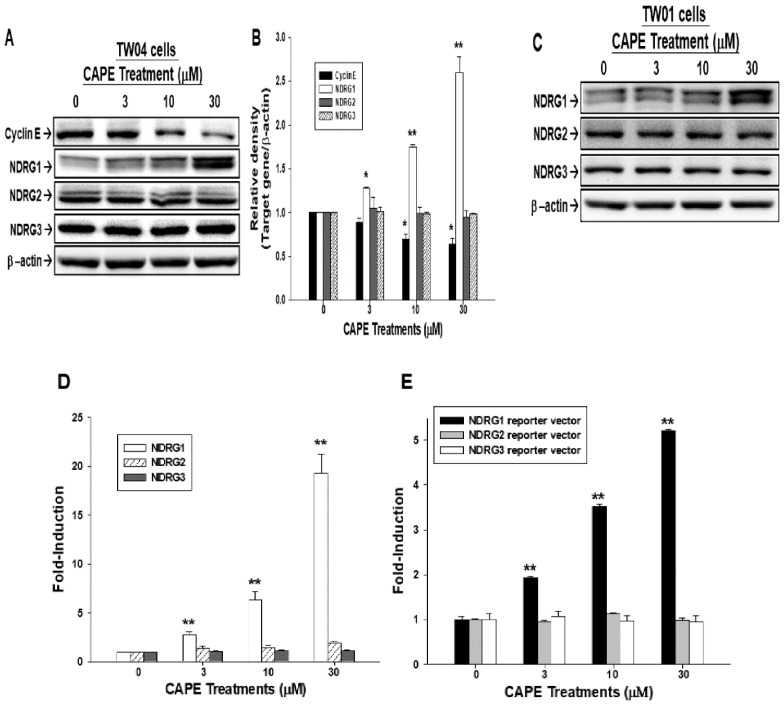
CAPE induces NDRG1 and cyclin E expressions in NPC cells. (**A**) TW04 cells were treated by CAPE in indicated concentrations for 24 h. The expressions of targeted proteins were determined by the immunoblot assay. (**B**) The quantitative data were expressed as the intensity of protein bands of the target genes/β-actin relative to the control solvent-treated group (*n* = 3). (**C**) The presentative immunoblot blot showing targeted proteins expressions in TW01 cells after indicated concentrations of CAPE treatment for 24 h. (**D**) Cells were treated with indicated concentrations CAPE for 24 h and the expression of the mRNA levels of targeted proteins were determined using RT-qPCR assays. Data were presented as mean fold-induction of the mRNA levels relative to the control solvent-treated group (±SE, *n* = 3). (**E**) The different report vectors were transfected into TW04 cells for 24 h, and cells were then treated by indicated concentrations CAPE for 24 h. Data were presented as the mean percentage of luciferase activity induced by the CAPE treatment relative to the control solvent-treated group (±SE, *n* = 6). (* *p* < 0.05, ** *p* < 0.01).

The result of the immunoblot assays illustrated that CAPE treatments significantly upregulated NDRG1 associated with the suppression of cyclin E protein levels in TW04 cells in a dose-dependent manner. 30 μM CAPE increased NDRG1 expression to 2.6-fold and decreased cyclin E expression to 0.7-fold (Figure 2A,B). However, CAPE treatments (3–30 μM) did not affect the expression of NDRG2 and NDRG3 (Figure 2A,B). A similar result was observed in TW01 cells, which showed only NDRG1 was stimulated by CAPE (Figure 2C). The RT-qPCR (Reverse transcription polymerase chain reaction) results showed that NDRG1 mRNA levels significantly increased after CAPE treatment in TW04 cells (Figure 2D). The promoter activity of NDRG1, but not NDRG2 and NDRG3, was also enhanced in TW04 cells treated with CAPE (Figure 2E). RT-qPCR and reporter assays showed the similar results with western blot.

### 2.3. NDRG1 Knockdown Enhances Cell Proliferation and Attenuates the Anti-Proliferation Effect of CAPE

**Figure 3:**
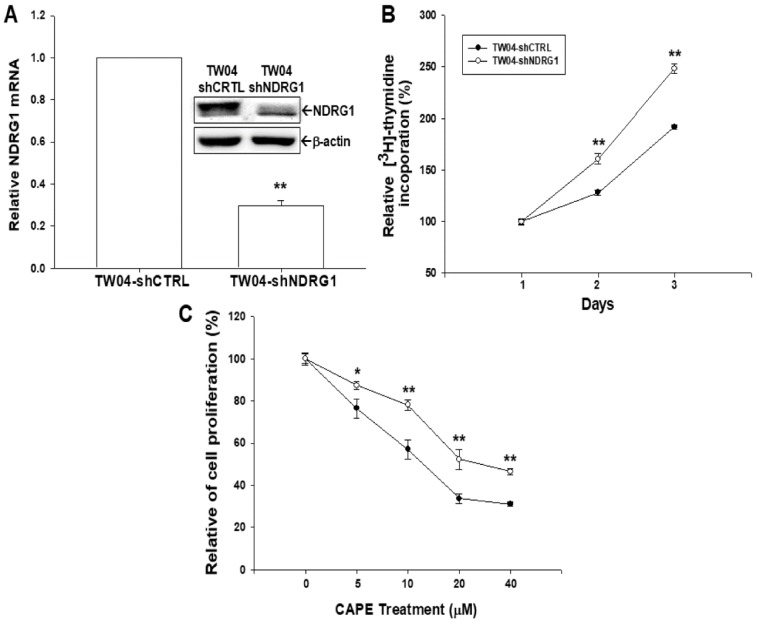
Knockdown of NDRG1 enhances cell growth in TW04 cell. (**A**) The expressions of NDRG1 in TW04-shCTRL and TW04-shNDRG1 cells were determined by immunoblot (top) and RT-qPCR (bottom) assays. (**B**) Proliferations of TW04-shCTRL (●) and TW04-shNDRG1 (○) cells were determined by the ^3^H-thymidine incorporation assay. The data were presented as the mean percentage of the TW04-shNDRG1 cells relative to the TW04-shCTRL cells (±SE, *n* = 6). The mean percentage (±SE) of cells in different days is compared to the day 1 (*n* = 6). (**C**) The TW04-shCTRL (●) and TW04-shNDRG1 (○) were treated with various concentrations of CAPE for 48 h, and growth inhibitory effect was determined by the CyQuant cell proliferation assay. The data were presented as the mean percentage (±SE) of cells relative to the solvent-treated control group (0 μM CAPE-treated, *n* = 8). (* *p* < 0.05, ** *p* < 0.01).

To evaluate the role of NDRG1 in NPC cell growth, we knocked down NDRG1 in TW04 cells (TW04-shNDRG1). The expressions of NDRG1 in the selected clones were determined by immunoblot (Figure 3A, top) and RT-qPCR (Figure 3A, bottom) assays. The result of ^3^H-thymidine incorporation assay revealed that TW04-shNDRG1 cells possessed much higher proliferative rate as compared to TW04-shCTRL (mock knockdown of NDRG1 TW04 cells) cells (Figure 3B). Results of CyQuant cell proliferation assay revealed TW04-shNDRG1 cells are less sensitive to CAPE treatment as compared to TW04-shCTRL cells (Figure 3C), implying CAPE represses TWO4 cells growth partly mediated by upregulating NDRG1 expression.

### 2.4. NDRG1 Knockdown Increases Cell Invasion in NPC Cells

**Figure 4:**
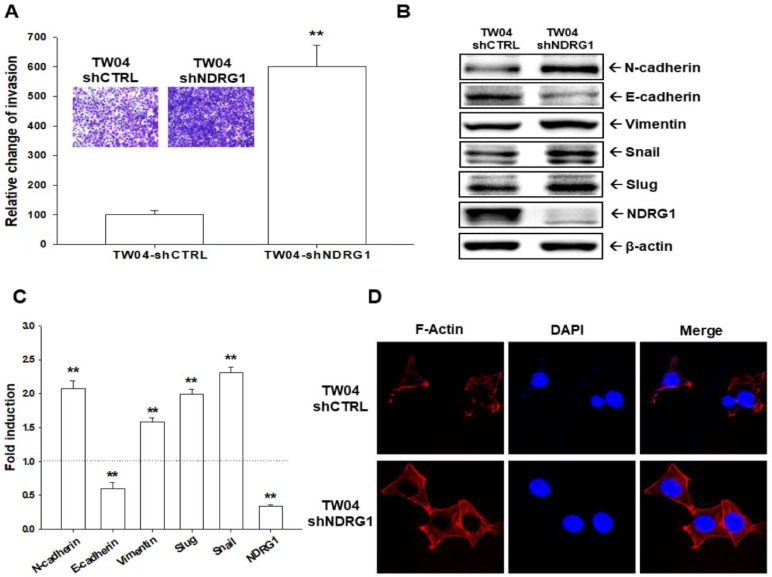
Knockdown of NDRG1 enhances cell invasion and induces epithelial–mesenchymal transition in TW04 cell. (**A**) The cell invasion of TW04-shCTRL and TW04-shNDRG1 were measured by the matrigel-invasion assay for 24 h incubation. The quantitative data were expressed as mean average (±SE, *n* = 6) relative to the TW04-shCTRL cells. (**B**) The expressions of targeted proteins in TW04-shCTRL and TW04-shNDRG1 cells were determined by the immunoblot assay. (**C**) The fold-induction data were expressed as the intensity of the protein bands from the target genes/β-actin relative to that of the mock-transfected cells (*n* = 3). (**D**) Distribution and intensity of F-actin (red) of TW04-shCTRL and TW04-shNDRG1 cells were determined by the immunofluorescence staining. DAPI (blue) was applied to stain the nucleus. (** *p* < 0.01).

To further evaluate the effect of NDRG1 on cell invasion in NPC cells, the matrigel invasion assay was applied and showed that knockdown of NDRG1 significantly enhanced the cell invasion in TW04 cells (Figure 4A, top). The quantitative analysis indicated that the invasion of TW04-shNDRG1 cells was significantly upregulated by 6-fold in comparison with the TW04-shCTRL cells (Figure 4A, bottom). The results from immunoblot assay (Figure 4B) and quantitative analysis (Figure 4C) showed that NDRG1 knockdown in TW04 cells significantly repressed the E-cadherin protein level but increased the levels of *N*-cadherin, Vimentin, Snail, and Slug proteins. Results of immunofluorescence staining with phallotoxin showed that NDRG1 knockdown in TW04 cells increased F-actin staining intensity at the leading edge of cells (Figure 4D).

### 2.5. CAPE Induces Phosphorylation of ERK, p38, and JNK in NPC Cells

**Figure 5:**
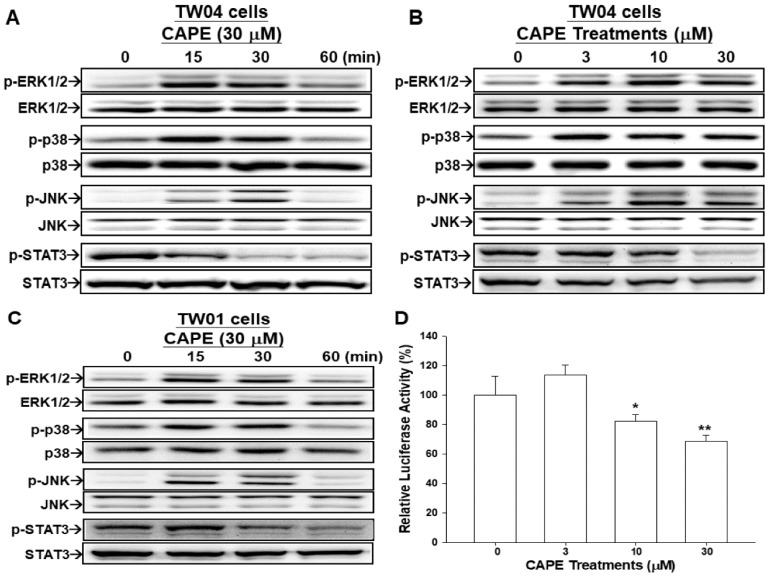
CAPE modulates phosphorylation of MAPK and STAT3 in NPC cells. (**A**) Time course of activities of components of MAPK signal pathway and STAT3 in TW04 cells treated with 30 μM CAPE. Expressions of ERK, p-ERK, p38, p-p38, JNK, p-JNK, STAT3, p-STAT3, and β-actin were determined by the immunoblotting assay. (**B**) Dose responses of ERK, p-ERK, p38, p-p38, JNK, p-JNK, STAT3, p-STAT3, and β-actin were determined by the immunoblotting assay after 15 min of CAPE treatments in TW04 cells. (**C**) Time course of activities of components of MAPK signal pathway and STAT3 in TW01 cells treated with 30 μM CAPE were evaluated by immunoblotting assay. (**D**) The pSTAT3-TA-Luc reporter vectors were transfected into TW04 cells for 24 h, and cells were then treated by indicated concentrations of CAPE for 24 h. Data were presented as the mean percentage of luciferase activity induced by the CAPE treatment relative to the control solvent-treated group (±SE, *n* = 6). (* *p* < 0.05, ** *p* < 0.01).

Results of the immunoblot assays showed that ERK, p38, and JNK were phosphorylated after 30 μM CAPE treatments in TW04 cells. The highest activation of JNK, ERK, and p38 was observed at 15 min after 30 μM CAPE treatment (Figure 5A). Interestingly, the immunoblot assays also showed a time-dependent inhibition of STAT3 phosphorylation after 30 μM CAPE treatment (Figure 5A). Further immunoblot assay illuminated a dose-dependent activation of ERK, p38, and JNK and inhibition of STAT3 phosphorylation in TW04 cells under the treatments of CAPE (3–30 μM) (Figure 5B). The similar finding was observed in TW01 cells as treated by 30 μM CAPE (Figure 5C). Results of reporter assays using the STAT3 specific reporter vector (pSTAT3-TA-Luc) containing the STAT3 binding site also indicated that CAPE downregulated STAT3 activity (Figure 5D).

### 2.6. CAPE Induces NDRG1 Expression via Phosphorylation of ERK, p38, and JNK in NPC Cells

**Figure 6:**
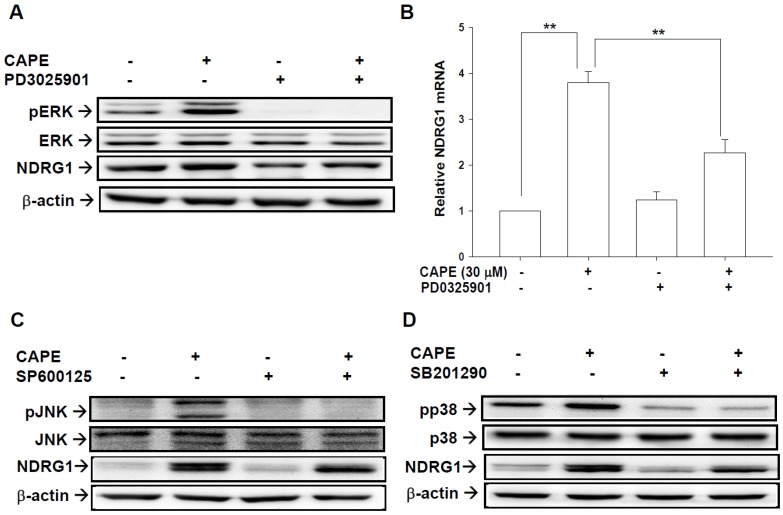
Inhibition of phosphorylation of MAPK blocks CAPE-enhanced expression of NDRG1 in TW01 cells. (**A**)The expression levels of ERK, p-ERK, NDRG1, or β-actin after pretreatments with (+) or without (−) PD0325901 for 1 h before 30 μM CAPE treatment in TW01 cells were determined by immunoblotting and RT-qPCR (**B**) assays. TW01 cells were pretreated with (+) or without (−) SP600125 (**C**) or SB202190 (**D**). The protein levels of JNK, p-JNK, p38, p-p38, NDRG1, or β-actin in CAPE-treated TW01 cells were determined by immunoblotting assay. (** *p* < 0.01).

To explore whether the enhancement of CAPE-induced NDRG1 expression in NPC cells was through the activation of MAPK pathway, we pretreated cells with MAPK elements inhibitors, ERK (PD0325901), p38 (SB202190), and JNK (SP600125), for 1 h before exposure to CAPE treatments. The result of immunoblot assay showed that the phosphorylation of ERK was blocked when cells were pretreated with PD0325901 in TW01 cells. The level of CAPE-induced NDRG1 protein was also inhibited by the pretreatment of PD0325901 (Figure 6A). Results of RT-qPCR showed the similar results (Figure 6B). The pretreatment of SP600125 (Figure 6C) or SB202190 (Figure 6D) inhibited not only the phosphorylation of JNK or p38, respectively, but also the CAPE-induced NDRG1 protein levels.

## 3. Discussion

CAPE, extracted from propolis, a natural health product derived from honeybee hives, has shown many pro-healthy effects, including anti-cancer effects. Thus, CAPE has been widely studied in the field of cancer treatment [15,21,22,23]. Our previous study in OSCC indicated that CAPE (5–30 μM) treatments or CAPE (10 mg/kg/day) intraperitoneal injection significantly attenuated cancer cell growth in vitro or in xenograft animal model [14]. Regarding NPC, CAPE-related studies are still very limited. Results of our study illuminated that CAPE was able to repress NPC cell growth in a dose-dependent manner and induced cell cycle arrest at S phase (Figure 1). Since metastatic NPC leads to a very poor prognosis, the finding that CAPE could attenuate NPC cell invasion further implies the great potential of CAPE for NPC treatment (Figure 1D). 

Cell cycle progression is necessary for cells to proliferate. In general, the cell cycle includes four phases, i.e., G1, S, G2, and M phase. Cell cycle progression is under strict control to maintain human body homeostasis. In cancer cells, the cell cycle is dysregulated due to constitutive mitogenic signaling stimulation, leading to uncontrolled cell growth [24]. Thus, targeting cell cycle progression has become one of the main stream cancer treatments. As shown in Figure 1A–C, CAPE inhibited TW04 cell growth through inducing S phase cell cycle arrest, which is supported by the finding that CAPE treatment downregulated cyclin E expression (Figure 2A,B) [25] and similar to the previous studies which showed CAPE could disturb cell cycle progression to slow cancer cell growth [6,10]. Our result is in line with our studies showing that CAPE treatments downregulated cyclin E expression in human prostate carcinoma PC-3 and overexpression of NDRG1 in human glioma U87 MG cells blocked cyclin E expression [26,27]. Whether the effect of CAPE on cyclin E expression depends on the NDRG1 requires further investigation.

NDRG1 has been widely deemed as a tumor suppressor gene in lots of cancers, including oral cancer [16,17,28,29,30]. Our group has shown for the first time that NDRG1 is also one of the CAPE downstream genes in oral cancer cells [14]. Previously, NDRG1 has been inferred as a tumor suppressor gene in NPC due to knockdown of NDRG1 promoted NPC cell proliferation, migration, and invasion of EBV-negative (Epstein-Barr virus) NPC 5-8F cells in vitro [20]. Kanda et al. also demonstrated that NDRG1 expression was less in NPC patients’ specimen [31]. Our result clearly showed that NDRG1, but not NDRG2 and NDRG3, was induced by CAPE in EBV-negative NPC TW04 cells in a dose-dependent manner, which was proved by western blot, RT-qPCR, and reporter assay (Figure 2) and consistent with our previous finding in oral cancer which showed only NDRG1 would be induced after CAPE treatment [14]. Previous study indicated that EBV-positive NPC C666-1 cells expressed extreme low levels of NDRG1 [31]; therefore, whether CAPE also affects cell proliferation, invasion, and tumorigenesis of EBV-positive NPC cells merits further studies. 

As we knocked down NDRG1 in TW04 cells, the cell proliferative rate increased, indicating NDRG1 is also a tumor suppressor gene in NPC cell, compatible with the previous study [20]. Furthermore, TW04-shNDRG1 cells exhibited less response to CAPE treatment (Figure 3C), suggesting CAPE repressed NPC cell growth partly through NDRG1 induction pathway. 

EMT is originally a vital process during embryogenesis [32] but latter to be found also as a key process for cancer progression and metastasis. During EMT, cancer cells gain mesenchymal cell markers, such as vimentin, α-smooth muscle actin (SMA), fibronectin, *N*-cadherin, etc. [33]. EMT is under strict control and well-orchestrated. Three families of transcription factors have been identified to induce EMT, including Snail/Slug, ZEB1/2, and Twist families [34]. 

Since EMT is a key process for tumor cells to metastasize to other places, adding the finding that TW04-shNDRG1 cells had greater invasive ability than TW04-shCTRL cells (Figure 4A), we further evaluated the influence of NDRG1 for EMT-related proteins. As shown in Figure 4B,C, NDRG1 knockdown increased Slug, Snail, Vimentin, and *N*-cadherin expressions and decreased E-cadherin expression, suggesting NDRG1 knockdown triggered EMT in NPC cells, leading to the increased invasiveness shown in Figure 4A, which was also supported by the increased F-actin synthesis after NDRG1 knockdown in TW04 cells (Figure 4D). These findings are consistent with the previous study showing NDRG1 attenuating EMT of NPC cells [20]. Although the precise mechanisms of NDRG1 effect on the EMT still need further investigation, however, previous study has suggested that NDRG1 blocked Smad2, but not Smad3, expression and phosphorylation to attenuate EMT process [20].

Regulation of the mitogen-activated protein kinase (MAPK) pathways has been identified as one of the anti-cancer mechanisms of CAPE in several cancers [14,35,36,37]. Results of this study revealed that CAPE-treated NPC cells had significantly increased phosphorylation of ERK, JNK, and p38 in a dose- and time-dependent manner (Figure 5A–C), which was consistent with previous studies showing the similar observations in C6 glioma and oral squamous carcinoma cells [14,37]. Our results demonstrated that the upregulating effect of NDRG1 by CAPE was attenuated by pre-treated with inhibitors of ERK (PD0325901), JNK (SP600125), or p38 (SB201290), respectively (Figure 6). However, unlike previous study of oral squamous carcinoma cells showing CAPE induced NDRG1 expression only through ERK signal pathway [14], the present study indicated that CAPE upregulated NDRG1 expression through multiple MAPK signal pathways in NPC cells since pretreatment of ERK, JNK or p38 inhibitor all partly blocked the effect of CAPE on NDRG1 induction.

Signal transducer and activator of transcription3 (STAT3) is a well-known transcription factor regulating many gene expressions involved in development and progression of various solid tumors [38]. Previous studies demonstrated that the overexpression and constitutive activation of STAT3 were involved in the oncogenesis of NPC cells, and treatment with STAT3 inhibitors suppressed the cell proliferation and tumor growth [39,40]. In this study, we found that CAPE treatments significantly decrease phosphorylation of STAT3 and blocked the STAT3 activity as shown by using the pSTAT3-TA-Luc reporter vector in TW04 cells (Figure 5). Our results indicated that CAPE treatment repressed NPC cell growth also partly mediated by repressing STAT3 phosphorylation.

## 4. Material and Methods

### 4.1. Cell Culture and Chemicals

Two EBV-negative NPC cell lines (keratinizing squamous TW01 and undifferentiated TW04 cells), which were derived from patients with NPC in Taiwan, were used in this study [41]. Cells were cultured in the RPMI-1640 medium (Life Technologies, Rockville, MD, USA) with 10% fetal calf serum (FCS; HyClone, Salt Lake City, UT, USA). Matrigel was purchased from Becton Dickinson Biosciences (Bedford, MA, USA). Bicinchoninic acid (BCA) protein assay kit was purchased from Pierce Protein Research (Rockford, IL, USA). CAPE, p38 inhibitor (SB202190), and ERK inhibitor (PD0325901) were purchased from Sigma-Aldrich Co. (St. Louis, MO, USA). JNK inhibitor II (SP600125) was purchased from Millipore (Temecula, CA, USA). CAPE was dissolved in DMSO at 60 μM in stock. All chemicals were dissolved in the suggested solvent by the manufacturer’s instructions.

### 4.2. Knockdown NDRG1

TW04 cells were transduced with NDRG1 small hairpin RNA lentiviral particles (Sc-3602-1-V; Santa Cruz Biotechnology, Santa Cruz, CA, USA) as previously described [16]. Briefly, cells were transduced with NDRG1 small hairpin RNA lentiviral particles as the NDRG1-knockdown (TW04-shNDRG1) cells. The mock-transfected TW04 cells (TW04-shCTRL) were transduced with control small hairpin RNA lentiviral particles (Sc-10808-V, Santa Cruz Biotechnology). Cells were selected with puromycin dihydrochloride after transduction.

### 4.3. Cell Proliferation Assay

Cells were seeded at 2000 cells/well in 96-well plates, the CyQuant cell proliferation assay kit (Invitrogen, Carlsbad, CA, USA) was used to measure the cell proliferation after treated with CAPE for 24 or 48 h. The cell proliferations of TW04 cells after mock-transfected or NDRG1 knock-down were measured using ^3^H-thymidine incorporation assay as previously described [16]. Each sample was tested in quadruplicate.

### 4.4. Flow Cytometry

TW04 cells were serum-starved for 24 h and then cultured in RPMI-1640 medium with 10% FCS and various concentrations of CAPE (0–30 μM) for another 24 h. Cell cycle analysis was performed using the FACS-Calibur Cytometer and CellQuestPro Software (BD Biosciences, San Jose, CA, USA), as previously described [42].

### 4.5. Immunoblot Assay

Equal quantities of cell extracts were separated on a 10% SDS-PAGE gel, transferred, and analyzed by the Western lightning plus-ECL detection system (Perkin Elmer, Inc., Waltham, MA, USA). Briefly, antibodies against NDRG1, NDRG2, NDRG3 (Invitrogen), Snail (Abcam, Cambridge, UK), cyclin E, E-cadherin, STAT3, N-cadherin, Vimentin (Abgent, San Diego, CA, USA), Slug, p44/42 MAPK, Phospho-p44/42 MAPK, SAPK/JNK, Phospho-SAPK/JNK, p38 MAPK, Phospho-p38 MAPK (Cell Signaling, Danvers, MA, USA), Phospho-STAT3 and β-actin (Millipore) were used and the protein bands were detected and quantified with the ChemiGenius Image Capture System (Syngene, Cambridge, UK) and the GeneTools Program of ChemiGenius (Syngene).

### 4.6. Real-Time Reverse Transcription-Polymerase Chain Reaction (RT-qPCR)

Total RNA from cells was isolated using Trizol reagent. The cDNA was synthesized, and real-time polymerase chain reaction (qPCR) was performed as previously described [14]. The mRNA levels were assayed using the FAM dye-labeled TaqMan MGB probes for human NDRG1 (Hs00608387_m1), NDRG2 (Hs0104515_m1), NDRG3 (Hs00259237_m1), and β-actin (Hs01060665_g1) were purchased from Applied Biosystems (Foster City, CA, USA). The transcript level of genes was normalized to β-actin levels.

### 4.7. Reporter Vector Constructs and Reporter Assay 

The human, NDRG1 (−4714 to +46), NDRG2 (−4253 to −1) and NDRG3 (−5734 to +178) reporter vectors were constructed as described in detail previously [14,43]. The reporter vector, pSTAT3-TA-Luc was purchased from Clontech Laboratories, Inc (Mountain View, CA, USA). TW04 cells were plated onto 24-well plates 1 day before transfection. Cells were then transiently transfected with luciferase reporter vector for additional 24 h after treated with or without CAPE and relative luciferase activities were then measured and reported in relative light units (RLU).

### 4.8. F-Actin Staining

The F-actin staining was performed as described previously [16]. Expression of F-actin in cells was revealed by incubation with Texas Red X-Phalloidin and mounted with ProLongR Gold reagent (Invitrogen). Immunofluorescence was examined using confocal microscope (LSM510 Meta, Zeiss, Oberkochen, Germany).

### 4.9. Matrigel Invasion Assay

The matrigel invasion assay for TW04-NDRG1si was performed as described previously [16]. For CAPE treatment, TW04 (1 × 10^5^) cells were treated with or without CAPE for 24 h, then the cells with or without CAPE were transferred to the matrigel-coated transmembrane for another 24 h. The images were captured using a digital camera connected to an inverted microscope (IX71, Olympus, Tokyo, Japan) with PAX-it Digital Image Management & Image Analysis and standardized for light intensity. 

### 4.10. Statistical Analysis

Statistical analyses were performed using SigmaStat program for Windows, version 2.03 (SPSS Inc. Chicago, IL, USA). Data were expressed as mean ±SE. The significance of differences was determined by Student’s *t*-test or one-way ANOVA with a *p* value less than 0.05 (* *p* < 0.05) or 0.01 (** *p* < 0.01).

## 5. Conclusions

Our results demonstrated that enhanced expression of NDRG1 partly contributed to the inhibitory effect of CAPE on cell proliferation and invasion in NPC cells. We provided evidence that NDRG1 knockdown enhanced cell proliferation, invasion, and EMT markers of NPC cells and rescued the suppressive effective of CAPE on NPC cell proliferation. This study is the first report to show CAPE induced NDRG1 expression through multiple MAPK signal pathways. We further demonstrated STAT3 activity was also repressed by CAPE in NPC cells. Since metastatic NPC patients have very poor prognosis, our results suggest CAPE could be a promising agent for NPC treatment. Further studies are justified.
